# Active View and Passive View in Virtual Reality Have Different Impacts on Memory and Impression

**DOI:** 10.3389/fpsyg.2019.02416

**Published:** 2019-11-01

**Authors:** Kyoko Hine, Hodaka Tasaki

**Affiliations:** ^1^Department of Computer Science and Engineering, Toyohashi University of Technology, Toyohashi, Japan; ^2^Department of Information Environment, Tokyo Denki University, Tokyo, Japan

**Keywords:** head-mounted display, active view, passive view, memory, perception, impression

## Abstract

Virtual reality (VR) through a head-mounted display (HMD) can provide new experiences. However, it remains unclear how the characteristics of HMDs affect users’ memory. To use HMDs more effectively and appropriately in several applied fields, including education, it is necessary to clarify what characteristics of HMDs affect users’ memory. A head-tracking function mounted on an HMD helps to detect the user’s head direction to enable a simulation experience akin to the real world. When we experience a simulation on an HMD, we actively perceive the visual world. In this study, we assessed how active/passive viewing affects users’ memory of VR content. We conducted a psychological experiment in which participants watched a movie on an HMD. In the active viewing condition, the presented view changed depending on the participant’s head direction. In the passive viewing condition, the presented view was a recorded movie that was shown to the participants in the active viewing condition. All participants took a memory test about the content presented in the movie on the day of viewing and 2 weeks later. The results showed that performance on the memory test in the active viewing condition was significantly lower than that in the passive viewing condition after 2 weeks. This result indicated that active viewing in VR inhibited users’ memory compared to passive viewing. The current study contributes to the development of new VR techniques, such as educational learning.

## Introduction

We can experience new worlds with new virtual reality (VR) technologies, such as head-mounted displays (HMDs). Recently, HMDs have gained popularity worldwide, and people can readily obtain new experiences *via* HMDs in several fields, including commercial and educational fields. HMDs are expected to become advanced media that can provide new experiences and a positive effect for users. For example, Martínez-Navarro et al. (2019) proposed a conceptual model in which HMDs can enhance a user’s memory of products. Another study argued that HMDs have high potential for use as an educational tool because HMDs offer experiences in VR similar to the real world, and this experience can enhance students’ learning (Freina and Ott, 2015). In addition, HMDs can improve concentration for learning content (Bodekær et al., 2016). Consequently, HMDs are increasingly used in classrooms to help learners understand and memorize effectively (Freina and Ott, 2015). However, few studies have assessed the effect of HMDs on users’ memory, and it remains unclear which characteristics of HMDs affect users’ memory in the real world *via* learning in VR. To use HMDs more appropriately and effectively in applied fields such as education, it is important to investigate which characteristics of HMDs affect users’ memory.

The head-tracking function is a signature technology of HMDs. A head-tracking function helps to detect the user’s head direction (Wang and O’hagan, 1998). HMDs can present various angles of view depending on the user’s head direction as detected by the head-tracking function. For example, when the head-tracking function detects a change in direction, HMDs change the view according to the user’s movement. This change evokes a special virtual experience that makes users feel as if they are moving in the real world.

When HMDs show the angle of view depending on the user’s head direction, the user actively perceives the visual world. Previous studies have revealed that active perception is important for appropriate responses to a given set of circumstances (Held and Hein, 1963; Mather and Lackner, 1980). Held and Hein (1963) showed that the performance of spatial perception tasks after active perception was higher than that after passive perception. Based on Held and Hein’s study, it is expected that spatial memory with active perception will be better than that with passive perception. This expectation has been supported by many studies, not only with regard to the spatial memory task (Herman and Siegel, 1978; Laudeman and Johnson, 1993; Larish and Andersen, 1995) but also with regard to memory tasks in other domains (Harman et al., 1999; Liu et al., 2007). However, the results of virtual environmental research are controversial. Péruch and Wilson (2004) reported that memory in the real world with learning through active viewing in a virtual environment was higher than memory with passive viewing, whereas other studies have reported no significant difference between active and passive viewing (Gaunet et al., 2001). Few studies have investigated the effect of active/passive viewing on memory using HMDs (Liu et al., 2011; Palermo et al., 2017), and the effect of active/passive viewing on memory using HMDs has not been directly assessed. Therefore, it is unclear whether the memory of VR content retrieved in the real world differs depending on active/passive viewing in the virtual world. In the current study, we investigated whether active/passive viewing *via* HMDs affects users’ memory of content in the real world.

**Figure 1:**
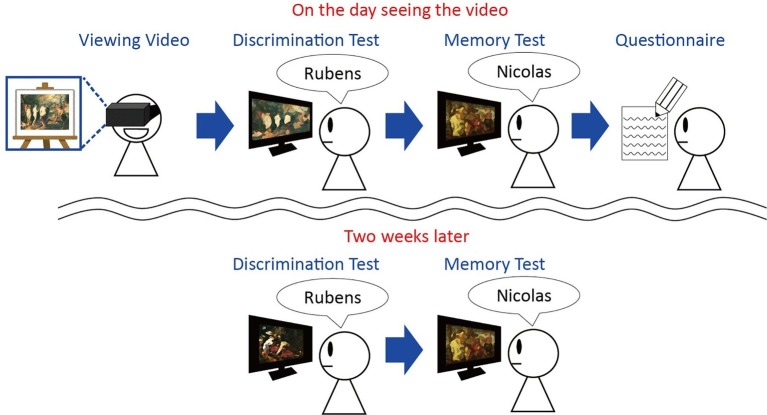
Experimental design. The participants watched the movie. The view of the movie presented in the active viewing condition changed with the participant’s head direction. The movie in the passive viewing condition presented recorded images from the active viewing condition. Then, all participants completed the discrimination test, the memory test and the questionnaire. Two weeks later, all participants completed the discrimination and the memory tests again. The paintings used in this study were from WikiArt (https://www.wikiart.org/en/nicolas-poussin/nurture-of-bacchus-1635; https://www.wikiart.org/en/peter-paul-rubens/the-judgment-of-paris-1636; https://www.wikiart.org/en/peter-paul-rubens/samson-and-delilah-1610; https://www.wikiart.org/en/peter-paul-rubens/rape-of-the-daughters-of-leucippus-1618). The examples in this figure have been modified. No further permission was required for the use of these images in the manuscript.

In the present study, we conducted a psychological experiment to assess how changes in active/passive viewing affect memory (Figure 1). Participants watched a 2D movie on an HMD in one of the two ways: active or passive viewing. In the active viewing condition, the presented view changed with the participant’s head direction. In the passive viewing condition, the presented view was a recording of the movie that was shown to the participants in the active viewing condition. While watching the movie, the participants learned about the paintings of two artists. Then, the participants completed a discrimination test and a memory test about the presented paintings in the movie, and they answered a questionnaire about the movie. The discrimination test asked the participants to distinguish between two painters’ paintings that had not been presented in the movie, whereas the memory test asked participants to distinguish paintings that had been presented. Two weeks later, all participants took the discrimination test and memory test again to assess the long-term effects. Based on a previous study that showed that active viewing memory enhanced visual memory (Péruch and Wilson, 2004), it was expected that performance on the memory test in the active viewing condition would be higher than that in the passive viewing condition. Additionally, it was expected that there would be no difference between the active and passive viewing conditions with regard to memory performance because some studies have not found an effect of active/passive viewing (Gaunet et al., 2001). For the discrimination test, participants were required to discriminate between paintings by Rubens and Nicolas that had not been presented. If the participants had formed concepts about Rubens’ and Nicolas’ paintings (Rubens and Nicolas styles), these concepts would help them select the correct answers in the discrimination test. Concept formation requires exposure examples (Bruner et al., 1956), and memory of examples affects concept formation (Weldon et al., 1989). Therefore, if the trials were sufficient to form concepts in this study, the participants’ performance in the active condition might be different from that in the passive condition when memory performance differed between the active and passive viewing conditions.

## Method

### Participants

We calculated the sample size with G*Power (Faul et al., 2007). A power analysis of repeated analysis of variance with G*Power recommended that the enrollment of 20 participants per group corresponded to an effect size of 0.20, a power of 0.85, and a *p* value of 0.05. Participants with normal vision and memory were recruited. Twenty participants (6 females and 14 males; aged 19–24, mean age = 21.05) were assigned to the active viewing condition, and 20 participants (3 females and 17 males; aged 19–24, mean age = 21.35) were assigned to the passive viewing condition. The participants were randomly assigned the two conditions. None of the participants had knowledge of Rubens’ and Nicolas’ paintings. All participants were informed in writing and signed a letter of consent. This study was approved by the Ethics Committee of Tokyo Denki University.

### Materials and Methods

#### Apparatus

Galaxy Gear VR^®^ (Samsung) and Galaxy S6^®^ (Samsung) were used to watch the movie. To control the HMD, we used screen mirroring software (MirrorOP Receiver^®^ and MirrorOP Sender^®^, MirrorOP). Additionally, we used recording software (Open Broadcaster Software studio^®^, OBS) to record the screen in the active viewing condition.

#### Paintings

Thirty Rubens paintings and 30 Nicolas paintings from WikiArt^1^ were used. The Rubens and Nicolas paintings were separated into three sets of 10 paintings. Each set of 10 paintings was used in the movie, the discrimination test and the memory test. The combinations of the sets of paintings and their use were counterbalanced.

#### Watching the Movie

**Figure 2:**
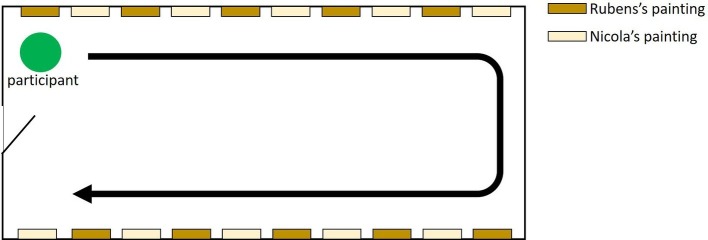
The floor plan of the room in the movie.

First, all participants watched the movie. The movie was recorded by RICHO THETA® as a panoramic video by two super-wide lenses. The views recorded by the two lenses were stitched. Then, the movie was attached to a 3D picture frame. The movie was presented in a sphere. The content included the introduction of the paintings and information about the painters. In the movie, the paintings were placed on the left and right walls in a rectangular room. Rubens’ paintings and Nicolas’ paintings were presented one by one. First, 10 paintings were placed on the left wall, and 10 pictures were placed on the right wall. Figure 2 shows the floor plan of the room in the movie. Each painting was presented for 10 s. In the active viewing condition, participants were able to look around freely in the movie, and the angle of viewing changed with the participant’s head movement. The screen of the HMD for the active viewing condition was recorded. In the passive viewing condition, participants viewed one of the recorded screens of the active viewing condition. Therefore, the angle of the view changed regardless of the participant’s head direction. To equalize the presented content for the active and passive viewing conditions, we used recorded video of the active viewing condition for the passive viewing condition. In both the active and passive viewing conditions, the movies presented for the right and left eyes were the same for each participant. In other words, all movies used in this study were 2D content, not 3D content, to avoid the effect of differences in binocular disparity among participants. The difference between the active and passive viewing conditions was whether the angle of viewing changed with the participant’s movement. The length of the movie was approximately 5 min. No instructions about physical movement were given; however, the viewing changed in correspondence with the participants’ head direction in the active viewing condition, and movement in a horizontal direction occurred automatically in the movie. In terms of changing the viewing angle in the movie, the average maximum changes for pitch, yaw, and roll were 19.4° (SD = 5.3), 38.8° (SD = 20.4), and 12.7° (SD = 7.5), respectively.

#### Discrimination Test

After watching the video, the discrimination test was conducted with a 20.1-inch display to assess the participants’ concept formation of the paintings. The distance between the computer display and the participant was approximately 35 cm. A fixation cross was presented in the center of the screen for 1,000 msec at the beginning of the trial and for 1,000 msec after a blank screen. Then, Rubens’ or Nicolas’ paintings were presented, and the participants identified the painter of the presented painting. The painting was presented until the participant provided a response. The total number of trials was 20. Half of the paintings were by Rubens, and the others were by Nicolas. None of the paintings were presented in the movie. If the participants had formed concepts about Rubens’ and Nicolas’ paintings, they could distinguish the paintings even though they had not previously seen the paintings. The order of the presentation was randomized.

#### Memory Test

Immediately after the discrimination test, the memory test was conducted. The procedure of the memory test was the same as that of the discrimination test, except that all paintings were presented in the movie. A memory test was conducted to assess the original memory. Therefore, the paintings used in the memory test were presented in the movie. In contrast, the discrimination test was conducted to assess whether the participants had formed concepts about Rubens’ and Nicolas’ paintings. Therefore, the paintings used in the discrimination test were not presented in the movie.

#### Questionnaire

At the end of the movie viewing, we investigated whether the participants’ impressions of the movie differed between the active and the passive viewing conditions by asking the participants to complete the questionnaire used in Iimura et al. (2012). The participants rated the following nine pairs on 7-point Likert scales: powerful-feeble, aggressive-calm, weak-strong, disgusting-comfortable, dislike-like, dark-bright, fake-real, wrong-accurate, or realistic-unrealistic. Additionally, the participants stated whether they watched a 2D or a 3D movie.

#### Tests After 2 Weeks

Two weeks later, all participants again completed a discrimination test and a memory test. The discrimination test was the same as the test conducted immediately after watching the movie, except the paintings were a new set that was not presented in the movie or the first discrimination test. The memory test was the same as the test conducted on the day the participants watched the movie. The discrimination test after 2 weeks was intended to evaluate whether the participants concepts of Rubens and Nicolas had been formed and preserved. The paintings used in the discrimination test were not previously used. In contrast, the paintings presented in the movie and the first memory test were used in the follow-up memory test to assess whether the original memory was preserved after 2 weeks. The order of the presentation of the paintings in the discrimination and memory tests was randomized. Finally, the participants were asked whether they had previously seen the paintings used in the study.

## Results

### Memory Test

**Figure 3:**
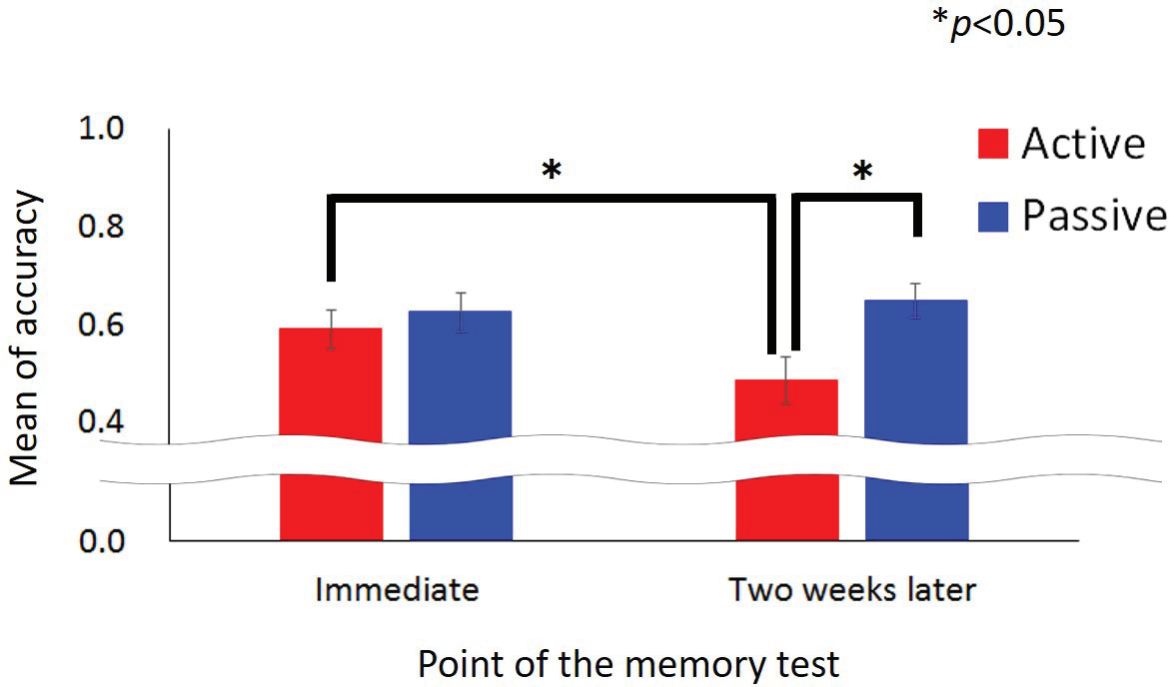
The mean accuracy of the memory test. The error bar indicates the standard error.

The accuracy of the memory test was calculated as the number of correct answers divided by the total number of items on the memory test. Figure 3 shows the mean accuracy of the memory test. For the immediate condition, the accuracy of the memory test was 0.59 (SD = 0.18) for the active viewing condition and 0.63 (SD = 0.18) for the passive viewing condition. For the two-week follow-up condition, the accuracy was 0.49 (SD = 0.22) for the active viewing condition and 0.65 (SD = 0.16) for the passive viewing condition. After ensuring the assumptions of normality and homoscedasticity, a two-way analysis of variance (ANOVA) was conducted for accuracy using the time of the test (immediate or 2 weeks later) and the viewing (active or passive) as factors. The main effect of the time was not significant, *F*(1,38) = 3.48, MSE = 0.19, *p* = 0.07, partial *η*^2^ = 0.08. The main effect of the view was also not significant, *F*(1,38) =2.11, MSE = 0.04, *p* = 0.15, partial *η*^2^ = 0.05. A significant interaction was found, *F*(1,38) = 4.13, MSE = 0.08, *p* = 0.04, partial *η*^2^ = 0.10. For the 2-week follow-up condition, the main effect of the view was significant, *F*(1,76) = 6.95, MSE = 0.25, *p* = 0.01, partial *η*^2^ = 0.15. However, the main effect of the view was not significant for the immediate condition, *F*(1,76) = 0.34, MSE = 0.01, *p* = 0.56, partial *η*^2^ = 0.01. For the active viewing condition, the main effect of the time of the test was significant, *F*(1,38) = 6.01, MSE = 0.11, *p* = 0.02, partial *η*^2^ = 0.15, whereas the main effect of the time was not significant for the passive viewing condition, *F*(1,38) = 0.17, MSE = 0.00, *p* = 0.68, partial *η*^2^ = 0.03.

### Discrimination Test

The accuracy of the discrimination test was calculated as the number of correct answers divided by the total number of items on the discrimination test. For the immediate condition, the accuracy of the discrimination test was 0.58 (SD = 0.18) for the active viewing condition and 0.59 (SD = 0.16) for the passive viewing condition. For the two-week follow-up condition, the accuracy was 0.50 (SD = 0.16) for the active viewing condition and 0.61 (SD = 0.16) for the passive viewing condition. After ensuring the assumptions of normality and homoscedasticity, a two-way ANOVA was conducted for accuracy using the time of the test (immediate or 2 weeks later) and the view (active or passive) as factors. The main effect of the time was not significant, *F*(1,38) = 2.07, MSE = 0.07, *p* = 0.16, partial *η*^2^ = 0.05 The main effect of the view was also not significant, *F*(1,38) = 0.67, MSE = 0.07, *p* = 0.42, partial *η*^2^ = 0.02. A significant interaction was not found, *F*(1,38) = 1.93, MSE = 0.05, *p* = 0.17, partial *η*^2^ = 0.05.

### 3D Perception

**Figure 4:**
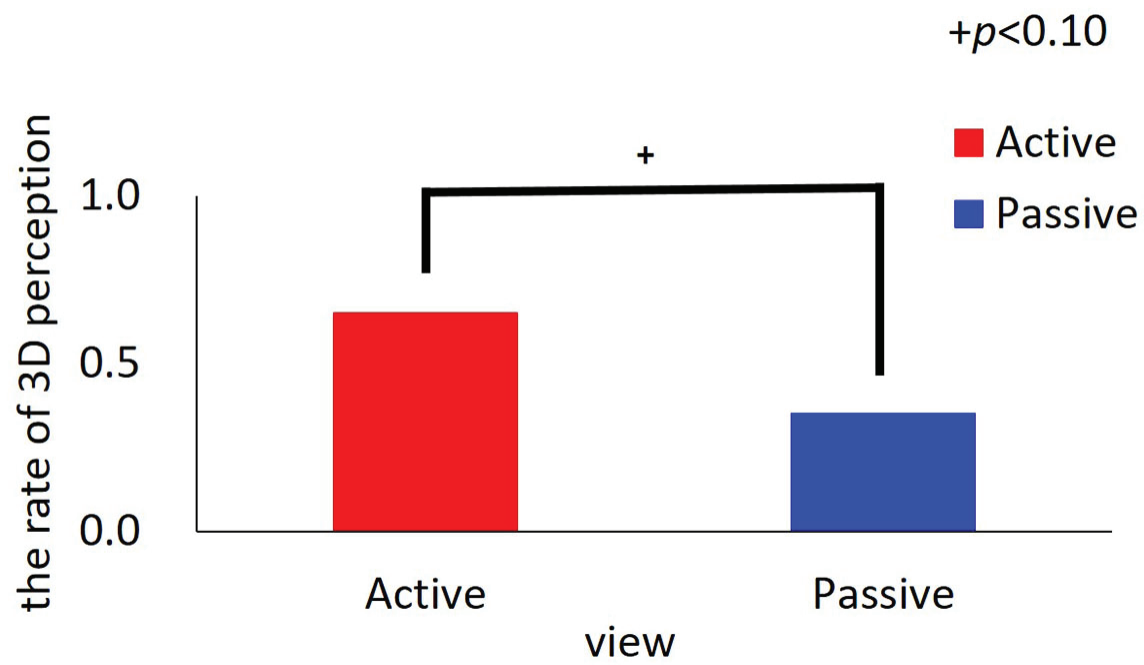
The rate of 3D perception.

The rate of the number of participants who answered that they watched the 3D movie was 0.65 for the active viewing condition and 0.35 for the passive viewing condition (Figure 4). There was a marginally comparable rate of 3D perception between the two groups [*χ*^2^(1, *N* = 40) = 3.60, *p* = 0.06, Cramer’s *V* = 0.30].

### Questionnaire

The semantic differential method was used to analyze the impression of the movie. The results of the questionnaire are presented in Table 1. A higher rating represents the impression of the left-side word for each pair of adjectives. Participants in the active viewing condition rated the movie as more powerful (*W =* 298, *p* = 0.01) and more realistic (*W* = 281.5, *p* = 0.05) compared to participants in the passive viewing condition.

**Table 1:** The median of the rating on the questionnaire.

	Median		*z*	*p*	Effect size (*r*)
	Active	Passive			
Powerful-feeble	5	3	2.71	0.00^**^	0.43
Aggressive-calm	5	4	1.42	0.16	0.22
Week-strong	5	3	1.75	0.08	0.28
Disgust-comfortable-	3	3	0.55	0.58	0.09
Dislike-like	5	4	1.56	0.12	0.25
Dark-bright	4	4	0.31	0.76	0.05
Fake-real	4.5	4	0.95	0.34	0.15
Wrong-accurate	4.5	4	1.13	0.26	0.18
Reality-unreality	5	3.5	2.23	0.03^*^	2.23

* *p < 0.05*.

** *p < 0.01*.

## Discussion

The aim of this study was to assess whether changing the active/passive view affects memory. We found that the participants’ performance on the memory test in the active viewing condition was lower than that in the passive viewing condition 2 weeks after viewing. There are some possible reasons for this result.

First, the reduction of memory performance in the active viewing condition might be caused by the effect of the environmental context on retrieval (Godden and Baddeley, 1980). Godden and Baddeley (1980) reported that memory performance when the context of retrieval is the same as the context of learning is better compared to when the context differs between retrieval and learning. In the current study, the room in which the memory test was conducted was the same as the room in which the participants watched the movie with the HMD. Participants in the active viewing condition might have experienced an entirely new world in the movie (i.e., they did not experience watching the movie in the experiment room but rather experienced seeing the paintings in another room), and the context of this new world differs from the situation of the memory test. On the other hand, participants in the passive view condition may have experienced watching the video in the same context as the memory test (i.e., they watched the movie in the experiment room). Therefore, the dissociation of the context between watching the movie and completing the memory test may have caused the reduction of memory performance in the active viewing condition compared to that in the passive viewing condition. This possibility has not yet been directly assessed. It has been reported that the congruency of the context is important to transfer learning from a virtual environment to the real world (Wallet et al., 2011). If the environmental context in VR influences memory performance in the real world, when the content in the VR is the same as the content of the real world, VR should provide an effective rehearsal and produce high performance. For example, performance in the real world should be enhanced by athletes’ rehearsal with VR.

Second, active viewing may not be suitable for learning about paintings. In this study, the participants in the active viewing condition rated the movie as more powerful and realistic than those in the passive viewing condition. In addition, the number of participants who perceived a 3D movie in the active viewing condition was larger than that in the passive viewing condition. These characteristics of perception may not have enhanced learning about the paintings in this study. Other learning that includes powerful and realistic impressions or 3D perception (e.g., carving learning, depth perception learning) might be enhanced by active viewing (i.e., VR). Few studies have systematically examined the effect of VR using HMDs and the relationship between 3D perception in VR and the types of remembered objects. Further studies should investigate which types of object memory are suitable for VR learning.

Third, the time the participants spent looking at the paintings in the active viewing condition may have been shorter than that in the passive viewing condition. In this study, the same movies were used in both the active and passive viewing conditions. Therefore, the length of time that the paintings were presented in the movies was the same in both conditions. However, the time that each participant spent looking at the paintings was not measured, and this time was not controlled in this study. It has been reported that longer viewing times promote memory performance (Laughery et al., 1971; Davies et al., 1977; Hall, 1980). Therefore, it could be expected that memory performance would be worse in the active viewing condition because the participants may have seen the paintings for less time compared to the participants in the passive viewing condition. Recently, HMDs that contain eye-tracking systems have been developed. The time a user spends looking at an object could be controlled by using such eye-tracking systems, which could clarify the effect of viewing time in VR on memory performance.

Fourth, head or body rotation might affect learning in virtual reality. The head or body rotation in the passive viewing condition may have been less than in the active viewing condition. If the head or body rotation in the active viewing condition was larger than that in the passive viewing condition, the participants in the active viewing condition may have experienced visual-induced sickness (Hettinger and Riccio, 1992), which could reduce the participants’ learning of the paintings. However, no participants left the experiment due to visual-induced sickness. In further studies, the impact of head and body rotation in VR on perception and memory should be systematically assessed.

One might think that the cognitive resources of the participants in the active viewing condition could have been assigned to sensory reception rather than to attention to the content. Sensory reception requires cognitive resources (Kihlstrom, 1987). As mentioned above, participants in the active viewing condition rated the movie as more powerful and realistic and tended to perceive a 3D movie. Their cognitive resources may have been involved in sensory reception rather than encoding the content of the movie; therefore, memory performance in the active viewing condition was lower than that in the passive viewing condition. Regarding spatial learning in virtual environments, Péruch and Wilson (2004) noted that passive viewing requires fewer cognitive resources, which might improve spatial learning. Recently, Makransky et al. (2019) reported an EEG study and suggested that high immersion in VR inhibited learning because of higher cognitive load. Although the use of cognitive resources alone could not fully explain the results of the current study because there was no difference in the memory test immediately after viewing, this possibility should be assessed in further studies.

Another possibility is that the novelty of the active viewing may have decreased the participants’ focus on the content of the movie. In the current study, most of the participants had not previously experienced VR (only one participant had experienced VR). Therefore, the active viewing with the HMD was the first experience of this kind for the participants, and they may have had more fun during the experiment than the participants in the passive viewing condition. Previous studies have indicated that HMDs are enjoyable (Roussou et al., 2006; Newbutt et al., 2016). In the current study, although there was no difference in the ‘dislike-like’ score between the active and passive viewing conditions, the score in the active viewing condition was higher than the score in the passive viewing condition. Previous research has reported that memory performance related to emotional content is better than memory performance related to neutral content (Kleinsmith and Kaplan, 1964). Two weeks after the first experience, participants in the active viewing condition recalled the emotion of their first experience (i.e., the VR experience) rather than the content of the movie. This may have caused the reduction in performance on the memory test in the current study. We should note that the enjoyment of a VR experience is different from the enjoyment of learning itself (learning about the paintings, in this study).

An additional interesting finding is that 3D perception occurred (65% in the active viewing condition) even though there was no binocular disparity. Binocular disparity is one of the factors that induce depth perception (Tsutsui et al., 2002). However, based on the results of the current study, binocular disparity is not a requirement for 3D perception. Further studies that investigate the role of active viewing with HMDs are required.

In the present study, the participants’ performance on the discrimination task did not differ between the active and passive viewing conditions. We used the discrimination task to assess concept formation. A concept is a mental category of objects or events and has a critical role in reducing information in cognitive processes and leading to appropriate behavior (Collins and Quillian, 1969). Concept formation requires exposure to many items to identify common features (Bruner et al., 1956). In our study, 10 paintings by Rubens and 10 by Nicolas were presented in the movie. More paintings could be presented to help participants more strongly form each concept. In fact, the participants’ performance on the discrimination test in the active viewing condition was lower than that in the passive viewing condition, although a significant difference was not found. This trend is the same as the result of the memory test. Further studies should clarify the effect of active/passive viewing on concept formation.

One of the limitations of the current study was the method of assessing memory. The participants’ performance on the memory and discrimination tests was assessed to evaluate the effect of active/passive viewing. Several factors may affect performance on paintings memory test, including memory ability (e.g., Burgess et al., 2002; Unsworth and Engle, 2007), knowledge (Ericsson and Charness, 1994), motivation (Weiner, 1966), and sleep (for long-term memory) (Stickgold, 2005; Marshall et al., 2006; Diekelmann and Born, 2010). The basic status of these factors was not assessed in this study. Therefore, these factors may have affected the results of the current study. Further studies should evaluate the profile of the participants with regard to these factors.

It has been recently suggested that HMDs be used in the classroom because HMDs provide better concentration for learning content (Bodekær et al., 2016). At this point, HMDs are a candidate for an educational tool. However, the current study suggests that VR experience may not completely support student learning. In line with the current study, Makransky et al. (2019) reported that instruction by VR using HMDs harmed students’ learning because of the features of immersive virtual reality compared to conventional media. To use HMDs more effectively, based on the results of the current study, specific content and instructions for VR should be offered to help students memorize more correctly and effectively. For example, when a specific object is to be memorized, the view in the HMD should not be changed regardless of the student’s movement. The characteristics of HMDs, such as active viewing, should be considered for use in applied fields.

## Conclusion

The current study showed that active viewing in VR inhibited memory performance after 2 weeks compared to passive viewing. There are several possible reasons for this result. Future studies should clarify why active viewing disrupts memory performance. To use HMDs more effectively in applied fields such as education, the content or instructions for VR should be offered with consideration of the characteristics of HMDs, that is, active viewing.

## Data Availability Statement

All datasets generated for this study are included in the article/supplementary material.

## Ethics Statement

The studies involving human participants were reviewed and approved by The Ethics Committee of Tokyo Denki University. The patients/participants provided their written informed consent to participate in this study.

## Author Contributions

HT designed the research and collected and analyzed behavioral data. KH designed the study, analyzed behavioral data and wrote the manuscript. All authors discussed the results and commented on the manuscript.

### Conflict of Interest

The authors declare that the research was conducted in the absence of any commercial or financial relationships that could be construed as a potential conflict of interest.

## Footnotes

^1^https://www.wikiart.org
